# Identification of a new mechanism for targeting myosin II heavy chain phosphorylation by *Dictyostelium *myosin heavy chain kinase B

**DOI:** 10.1186/1756-0500-3-56

**Published:** 2010-03-03

**Authors:** Julie Underwood, Jonathan Greene, Paul A Steimle

**Affiliations:** 1 Department of Biology, University of North Carolina at Greensboro, Greensboro, North Carolina, USA

## Abstract

**Background:**

Heavy chain phosphorylation plays a central role in regulating myosin II bipolar filament assembly in *Dictyostelium*, as well as in higher eukaryotic nonmuscle cells. Our previous work has demonstrated that the WD-repeat domain of *Dictyostelium *myosin II heavy chain kinase B (MHCK-B), unlike its counterpart in MHCK-A, is not absolutely required for targeting of the kinase to phosphorylate MHC. Thus, we tested the hypothesis that an asparagine-rich and structurally disordered region that is unique to MHCK-B can by itself function in substrate targeting.

**Findings:**

Biochemical assays comparing the activities of full-length MHCK-B, a truncation lacking only the WD-repeat domain (B-Δ-WD), and a truncation lacking both the N-rich region and the WD-repeat domain (B-Δ-N-WD) revealed that the N-rich region targets MHCK-B to phosphorylate MHC in a manner that leads to bipolar filament disassembly. This targeting is physiologically relevant since cellular over-expression of the B-Δ-WD truncation, but not the B-Δ-N-WD truncation, leads to dramatically reduced levels of myosin II filament assembly and associated defects in cytokinesis and multicellular development.

**Conclusions:**

The results presented here demonstrate that an intrinsically unstructured, and asparagine-rich, region of a MHCK-B can mediate specific targeting of the kinase to phosphorylate myosin II heavy chain. This targeting involves a direct binding interaction with myosin II filaments. In terms of regulating myosin bipolar filament assembly, our results suggest that factors affecting the activity of this unique region of MHCK-B could allow for regulation of MHCK-B in a manner that is distinct from the other MHCKs in *Dictyostelium*.

## Background and Hypothesis

Myosin II is a molecular motor that plays a central role in facilitating a broad range of cellular activities in nonmuscle cells by driving contraction of actin filaments. In nonmuscle cells, myosin II exists in a dynamic equilibrium between bipolar filaments that can contract apposing actin filaments and monomers that are contraction incompetent. Studies in *Dictyostelium discoideum *[1], and more recently in mammalian nonmuscle cells [2], have demonstrated that phosphorylation of regulatory sites in the "tail" region of the myosin II heavy chain (MHC) drive bipolar filament disassembly. MHC phosphorylation in *Dictyostelium *is catalyzed by at least three MHC kinases (MHCK-A, -B, and -C) that share homologous α-kinase and WD-repeat domains [3]. We have shown previously that the WD repeat domain is involved in physically targeting the catalytic domains of MHCK-A and MHCK-B to phosphorylate myosin II substrate [4]. Even so, a truncation of MHCK-B lacking its WD-repeat domain, unlike the analogous truncation of MHCK-A, still phosphorylates myosin II up to 20% of the level observed with the full-length kinase [4]. This suggests that there are additional mechanisms by which MHCK-B-mediated phosphorylation of MHC can be achieved.

A potentially relevant structural difference between the MHCK-A and -B proteins is that MHCK-B possesses a region of 125 amino acids between its catalytic and WD-repeat domains that is predicted to exhibit a high level of structural disorder [5]. This region is strikingly enriched in asparagine residues (28% of the amino acids) with a stretch of 26 asparagines interrupted by a single serine residue. From this point forward we will refer to this region as the N-rich (asparagine-rich) region of MHCK-B. In the studies presented here, we extend the results from previous studies [4,6] by exploring the hypothesis that the N-rich region of MHCK-B plays a role in facilitating WD-repeat-independent phosphorylation of MHC by the kinase. A broader goal of these studies is to examine the potential for an inherently unstructured region of a protein to play a role in substrate targeting.

## Methods

### Cell Culture and Cell-Based Assays

*Dictyostelium *cells were cultured as described previously [7] in HL5 supplemented with penicillin and streptomycin. Cell lines harbouring recombinant expression plasmid were selected at 50 μg/ml Geneticin (G418) for fusion protein over-expression. Cell lines over-expressing full-length or truncated versions of MHCK-B were analyzed for defects in cytokinesis and multicellular development as described previously [6]. Cell lines were analyzed for their levels of myosin II filament assembly as described by Rico and Egelhoff [6], with the exception that the MHCK-B proteins were over-expressed in a *mhkA/B/C*-null background [8]. AX2, *mhkB*-null [6], and *mhkA/B/C*-null cell lines were obtained from the Dicty Stock Centre [9].

### Fusion Protein Construction, Expression, and Purification

**Figure 1:**
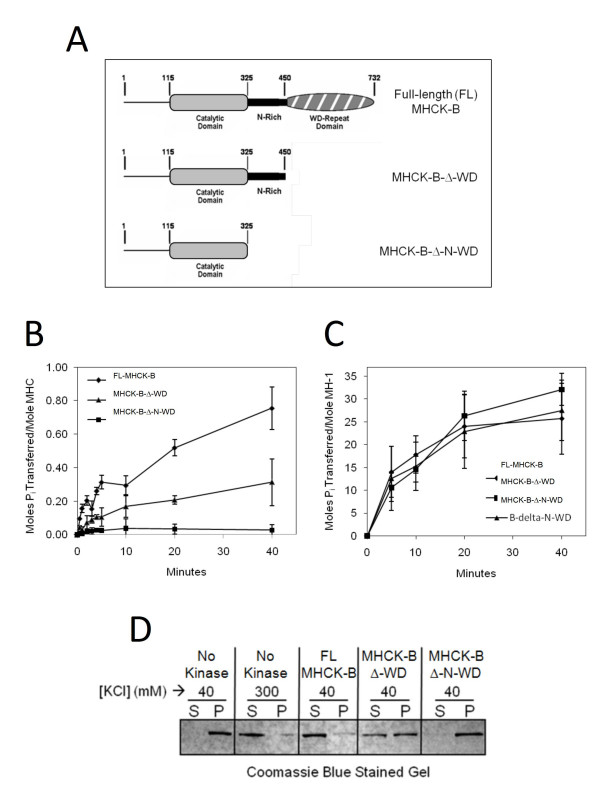
**Comparison of the kinase activities of MHCK-B and its truncations toward myosin II and MH-1 peptide substrate**. (A) Illustrations of the domain organization of MHCK-B and its truncations used in these studies. (B) Kinase assays containing 50 nM purified GST-MHCK-B (◆), GST-B-Δ-WD (▲), or GST-B-Δ-N-WD (■) and 1.0 μM purified *Dictyostelium *myosin II were performed as described previously [4]. (C) The same proteins (50 nM) were also assessed for MH-1 peptide (50 μM) phosphorylation over time [4]. (D) Coomassie stained SDS-PAGE gel showing MHC in pellet (P) and supernatant (S) fractions. The assembly of *Dictyostelium *myosin II (800 nM) was assessed as described previously [6] either after mock phosphorylation (untreated) or after phosphorylation for 15 min with 80 nM kinase. After phosphorylation, samples were adjusted to 40 mM NaCl to optimize filament assembly and then subjected to centrifugation to pellet assembled myosin. For graphs (B) and (C), each plotted point represents the average value from three separate experiments and the vertical lines are the standard errors of those means.

*Dictyostelium *cells expressing Flag-tagged full-length MHCK-B have been described previously and were a generous gift from the laboratory of Tom Egelhoff (Cleveland Clinic Foundation - Department of Cell Biology) [6]. The generation of *Dictyostelium *cells expressing Flag-tagged MHCK-B truncations followed the strategy described in detail previously [7], starting with PCR amplification of each MHCK-B truncation using plasmid-cloned full-length *mhkB *as template [6] and in frame primers containing *Bam*HI restriction enzyme sites for eventual transfer into the *Dictyostelium *expression vector pTX-Flag [10]. All amino acid numbering for MHCK-B in this text refers to the GenBank™ protein entry [GenPept:XP_636368]. Generation of the MHCK-B truncation lacking only the WD-repeat domain (designated B-Δ-WD) involved PCR amplification of the *mhkB *gene encoding amino acids 1 - 450, whereas the region encoding amino acids 1 - 325 was amplified to generate a truncation lacking both the N-rich and WD-repeat domains (designated B-Δ-N-WD), and thus contains only the catalytic and amino-terminal regions of the protein (see Figure 1A for schematic of MHCK-B truncations). Recombinant vectors were introduced into *mhkB*-null *Dictyostelium *cells as described previously [7]. The B-Δ-N-WD PCR amplification product was also inserted into the pGEX-4T vector (GE Life Sciences) for bacterial expression as a GST-fusion protein. The expression and purification of all GST-fusion proteins used in these studies were performed exactly as described previously for MHCK-B (full-length) and MHCK-B-Δ-WD [4].

### Kinase Phosphorylation Assays, Myosin II Assembly Assays, and Myosin II Co-Sedimentation Assays

Phosphorylation assays for MHC, MH-1 peptide, and myelin basic protein (MBP) were performed as described previously, except that kinase was added at 50 nM [4] and the concentration of myosin II included in the assay was 1.0 μM instead of 0.42 μM as in previous studies. Myosin II assembly assays were performed exactly as detailed by Rico and Egelhoff [6], except that kinase was added at 80 nM. Experiments analyzing MHCK-B fusion proteins for co-sedimentation with myosin II filaments were carried out as described previously [4].

## Results

### The N-Rich Region Facilitates MHCK-B Phosphorylation of MHC In Vitro

In the current studies, we tested the hypothesis that the N-rich region of MHCK-B is able to function in substrate targeting. If this is the case, then the ability of an MHCK-B truncation lacking both the N-rich and WD-repeat domains (B-Δ-N-WD) to phosphorylate MHC will be greatly reduced or absent. To explore this possibility, purified GST-tagged MHCK-B (full-length), B-Δ-WD, and B-Δ-N-WD proteins (Figure 1A) were assayed for kinase activity toward *Dictyostelium *MHC, as well as toward a peptide substrate (MH-1). MH-1 has been shown previously to be phosphorylated by alpha kinase catalytic domains in a WD-repeat-independent manner [4], and thus its phosphorylation is a useful measure of the basal kinase activity of the catalytic domain.

We found that removal of both the N-rich and WD-repeat domains (GST-B-Δ-N-WD) renders the catalytic domain barely able to phosphorylate MHC above detectable levels, whereas the truncation containing the N-rich region (GST-B-Δ-WD) can still use MHC as a substrate, albeit at about 30% of that displayed by the full-length kinase (Figure 1B). Taken together, these results suggest that removal of the N-rich region severely compromises MHC phosphorylation by the catalytic domain. By contrast, the innate kinase activity of the catalytic domain is not lost upon removal of the N-rich region and/or the WD-repeat domain of MHCK-B since all three versions of MHCK-B phosphorylated MH-1 peptide to the same level (Figure 1C). Moreover, we found that the presence of the N-rich region has no effect on the phosphorylation of another protein substrate, MBP (Additional file 1), suggesting that the targeting activity of the N-rich region is specific for MHC.

**Figure 2:**
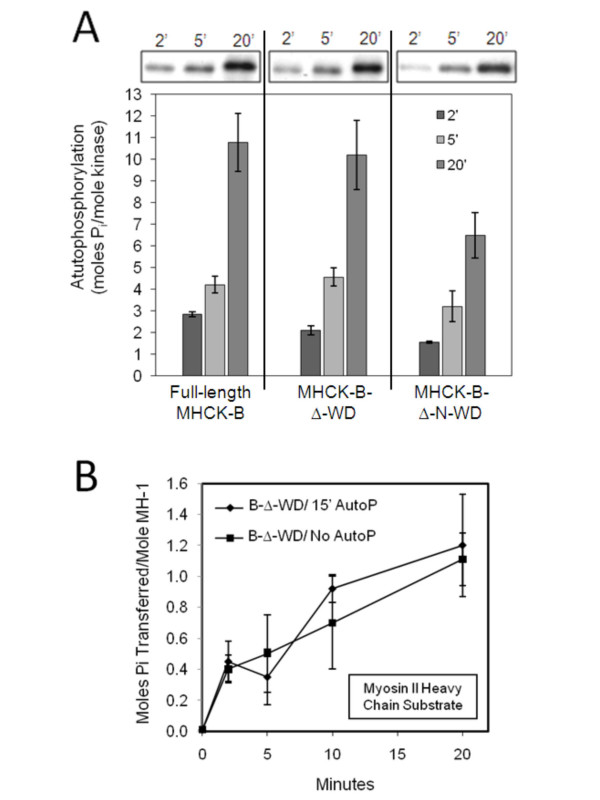
**Analysis of autophosphorylation of MHCK B and its truncations and the effect on kinase activity**. (A) Autophosphorylation reactions were performed at 200 nM of each fusion protein, and level of autophosphorylation was determined as described previously [15] by subjecting aliquots of autophosphorylation reactions at 2 min, 5 min, and 20 min to SDS-PAGE, Coomassie Blue staining, and then scintillation counting of excised kinase bands. Visual analysis of autophosphorylation (above bar graph) was achieved via autoradiography of dried, Coomassie-stained SDS-polyacrylamide gels of autophosphorylation time points. The bars represent the average values from at least three separate experiments and the vertical lines are the standard errors of those means. (B) Pre-autophosphorylated GST-B-Δ-WD (50 nM) was compared with control (not pre-autophosphorylated) fusion protein for its ability to phosphorylate MHC. Kinase assays were performed as described previously (Figure 1B) and the activities of pre-autophosphorylated GST-B-Δ-WD (◆, "B-Δ-WD/15'AutoP") and control GST-B-Δ-WD (■, "B-Δ-WD/No AutoP") were measured over time. Each plotted point represents the average value from three separate experiments and the vertical lines are the standard errors of those means.

Further analyses revealed that the B-Δ-N-WD truncation exhibited reduced levels of autophosphorylation compared to the full-length kinase and the B-Δ-WD truncation, (Figure 2A). This suggests that a portion of the 15 to 20 autophosphorylation sites in MHCK-B [4,6] reside in the N-rich region of the kinase. A recent study of the mammalian alpha-kinases TRPM6/TRPM7 revealed that the ability of these kinases to phosphorylate myosin II heavy chain is dependent on the autophosphorylation of unstructured regions of these kinases [11]. We examined the B-Δ-WD truncation for a similar mode of regulation and found that pre-autophosphorylation had no apparent effect on the ability of the truncation to phosphorylate MHC substrate (Figure 2B). This result is consistent with previous studies demonstrating that autophosphorylation of full-length MHCK-B has no effect on the kinase activity of the enzyme [6].

### Targeting by the N-Rich Region Leads to Myosin II Filament Disassembly In Vitro

**Figure 3:**
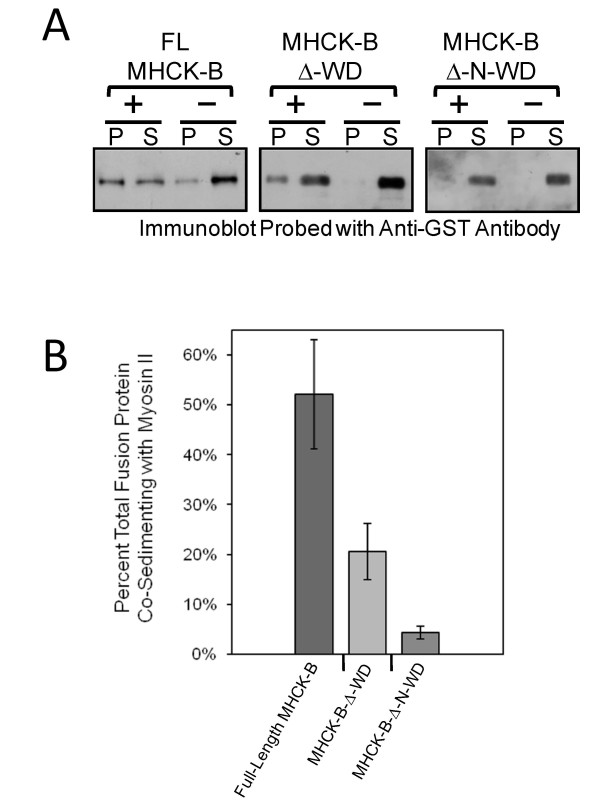
**Comparison of MHCK-B and its truncations for co-sedimentation with myosin II bipolar filaments**. (A) Kinase fusion proteins (0.1 μM) were incubated with myosin filaments (1.0 μM) as described previously [4]. Reaction mixes were centrifuged and equal volumes of the resulting pellets (P) and supernatants (S) were subjected to SDS-PAGE and the kinase fusion proteins were identified by Western blotting with anti-GST antibody. (B) Bar graph of kinase constructs binding to myosin II. Co-sedimentation values were quantified by densitometric analysis of the Western blot, and the amount of fusion protein in the pellet fraction was divided by that in both the pellet and supernatant and multiplied by 100%. The bars represent the average values from at least three separate experiments and the vertical lines are the standard errors of those means.

Our results thus far demonstrate that the N-rich region alone can serve as a MHC targeting domain; however, it is not clear if MHC phosphorylation via this mechanism indeed drives myosin II filament disassembly [12]. To explore this possibility, we examined the ability of purified myosin II to assemble into sedimentable filaments after phosphorylation by GST-tagged MHCK-B or its truncations. We found MHC phosphorylation by both MHCK-B (full-length) and MHCK-B-Δ-WD promotes myosin II filament disassembly, whereas incubation with MHCK-B-Δ-N-WD has no effect on the ability of myosin II to form filaments (Figure 1D). We have shown previously that removal of the WD-repeat domain of MHCK-B reduces the ability of the kinase to interact directly with myosin II filaments to about 30% of that exhibited by the full-length kinase [4]. In the current study, we found that the B-Δ-N-WD truncation did not co-sediment with myosin II filaments (Figure 3A and 3B), suggesting that the N-rich region facilitates phosphorylation of MHC by binding directly to myosin II filaments.

### Cells Over-Expressing MHCK-B-Δ-WD Exhibit Cytokinesis Defects and Decreased Myosin II Assembly

**Figure 4:**
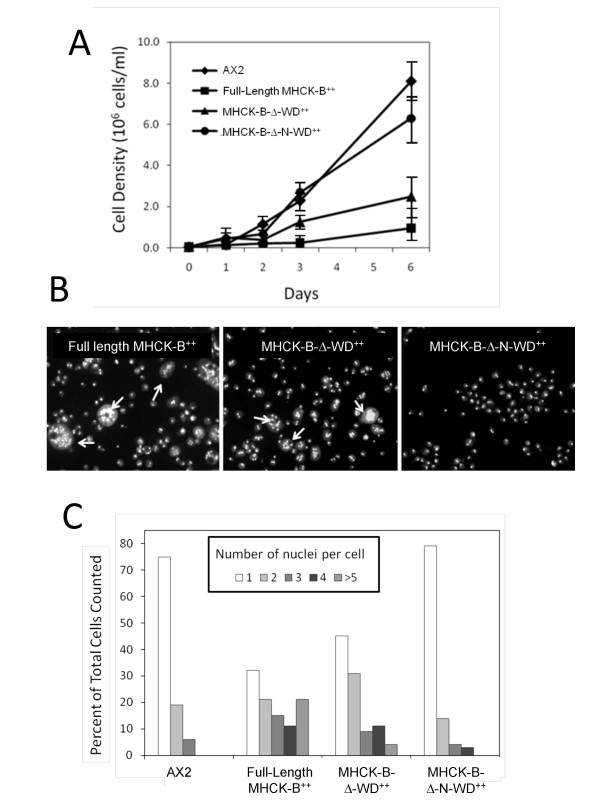
**Analysis of suspension growth and multinuclearity of cells over-expressing full-length MHCK-B, MHCK-B-Δ-WD, or MHCK-Δ-N-WD**. (A) Cell densities were determined for wild type AX2 cells (◆), or for cells over-expressing full-length MHCK-B (■), the MHCK-B-Δ-WD truncation (▲), or the MHCK-B-Δ-N-WD truncation (●). Cells were grown in suspension culture (HL5 medium, 175 rpm shaking, 25°C) and cell densities were determined on the days indicated on the x-axis. Each plotted point represents the average value from four separate experiments and the vertical lines are the standard errors of those means. (B) Epifluorescent images of DAPI stained cells from the indicated cell lines were taken after four days of growth in suspension using an Olympus IX70 microscope system and an UPlanFL 20× objective lens. (C) The number of nuclei/cell was determined for each of the cell lines after four days of growth in suspension. The number of nuclei per cell was determined for a total of 250 cells per cell line over two separate experiments.

We next tested the hypothesis that if substrate targeting by the N-rich region is physiologically significant, then over-expression of the MHCK-B-Δ-WD truncation in *Dictyostelium *cells should lead to an increase in the amount of cellular myosin II in the disassembled state. In turn, myosin II dependent processes, such as cytokinesis in suspension culture and multicellular development, should be compromised [3]. To this end, we compared the suspension culture growth rates of *Dictyostelium *cells over-expressing full-length MHCK-B, MHCK-B-Δ-WD or MHCK-B-Δ-N-WD (Additional file 2) with that of wildtype AX2 cells. Indeed we found that cells over-expressing MHCK-B-Δ-WD proliferate in suspension culture at a much slower rate than AX2 cells and become increasingly large and multinucleated over time (Figure 4A, B, and 4C). By contrast, cells over-expressing the MHCK-B-Δ-N-WD truncation grow normally in suspension culture. Likewise, we found that cells over-expressing either full-length MHCK-B or the B-Δ-WD truncation stalled at the mound stage of multicellular development, whereas those with elevated levels of the B-Δ-N-WD truncation completed the developmental cycle normally (Additional file 3).

**Figure 5:**
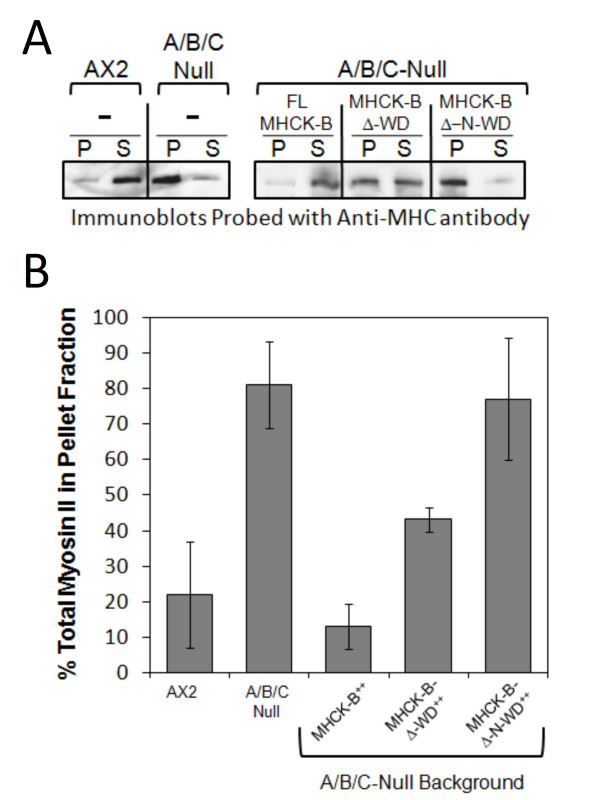
**Comparison of the level of myosin II assembly in cells over-expressing full-length MHCK-B, MHCK-B-Δ-WD, or MHCK-Δ-N-WD**. (A) Immunoblot of pellet (P) and supernatant (S) fractions from cytoskeletal fractionation assays performed as described previously [6,13]. The background *Dictyostelium *cell lines are indicated above each bracket and the over-expressed protein is indicated within the brackets and above each corresponding P and S pair. Myosin II heavy chain was detected in the fractions using polyclonal antibody specific the heavy chain. (B) The relative amount of myosin II (244 kDa MHC band) in the P and S fractions was quantified desnsitometrically from Western blots as described previously [6,13], and the percent total myosin II in the cytoskeleton-enriched fraction was calculated by dividing the densitometry value for the P fraction by the sum of those for the P and S fractions and then multiplying by 100 percent. The bars represent the average values from four separate experiments and the vertical lines are the standard errors of those means.

The cellular defects observed with B-Δ-WD over-expression are consistent with a decreased ability of the cell to form myosin II bipolar filaments. To explore this possibility we examined the assembly state of cellular myosin II by analyzing the levels of myosin II associated with the detergent-insoluble fraction of cells over-expressing full-length MHCK-B or its truncations. These experiments were performed as described previously [6,8,13] except that the MHCK-B proteins were over-expressed in the *mhck A/B/C-null *background [8], as a means of increasing the sensitivity of this assay. In these cells, myosin II is constitutively over-assembled due to the absence of MHCK-A, -B, and -C activities [8] (Figure 5A). As a result, decreased assembly of myosin II filaments is more evident than in AX2 cells where the level of cytoskeleton associated myosin II is relatively low in vegetative cells. We found that over-expression of the full-length or B-Δ-WD versions of MHCK-B in the *mhck A/B/C-null *background resulted in an approximately 85% and 46% reduction in the amount of assembled myosin, respectively (Figure 5A and 5B). By contrast, over-expression of the B-Δ-N-WD truncation did not lead to a decrease in the level of myosin II associated with the cytoskeleton-enriched pellet of the cell (Figure 5A and 5B); thus indicating that the N-rich region can target the MHCK-B catalytic domain to phosphorylate MHC and drive filament disassembly in the cell.

## Conclusions

The results presented here extend our previous studies of WD-repeat domain mediated targeting of enzyme activity [4] and have revealed that a highly disordered [5] and asparagine-rich region of MHCK-B can guide the catalytic domain to phosphorylate MHC and drive myosin II filament disassembly The demonstrated importance of the N-region in defining the catalytic activity of MHCK-B suggests that factors targeting this unique region could provide a means of regulating the kinase in a manner that is distinct from the other MHCKs in *Dictyostelium*. In a broader context, our findings support the idea that highly specific substrate targeting can be mediated by a region of an enzyme that lacks a recognizable motif or predicted fold. These findings may be of particular significance to studies of other *Dictyostelium *proteins in which asparagine-rich regions are fairly common, but their functions are largely unknown [14].

## Competing interests

The authors declare that they have no competing interests.

## Authors' contributions

JU performed most of the kinase assays, myosin II sedimentation experiments, and growth curve experiments. JU also prepared the recombinant vectors for expression of GST-tagged and Flag-tagged MHCK-Δ-N-WD fusion proteins. JU affinity purified all of the GST-fusion proteins from bacterial cells. JG performed the Triton-X100 fractionation studies and performed some of the kinase assays. PAS conceived of the study, participated in its design and coordination, and trained JU and JG in the techniques required to perform the experiments, and contributed to the execution of the kinase and growth curve experiments. PAS wrote the manuscript. JU and JG read and approved the final manuscript.

## Supplementary Material

Additional file 1 **Analysis of MHCK-B truncations for phosphorylation of myelin basic protein**. Plots of myelin basic protein phosphorylation by full-length MHCK-B, MHCK-B-Δ-WD, and MHCK-B-Δ-N-WD over time. Click here for file

Additional file 2 **Analysis of the expression levels of MHCK-B, MHCK-B-Δ-WD, and MHCK-B-Δ-N-WD in Dictyostelium cells**. Immunoblots of cell lysates from AX2 cells (endogenous MHCK-B) and cells over-expressing MHCK-B, MHCK-B-Δ-WD, or MHCK-B-Δ-N-WD. Bar graph of the level of over-expression as determined by densitometric analysis of bands in the immunoblots. Click here for file

Additional file 3 **Multicellular development of cells over-expressing full-length MHCK-B, MHCK-B-Δ-WD, or MHCK-B-Δ-N-WD**. Digital images of the progress of *Dictyostelium *multicellular development after five days under starvation conditions. Click here for file
