# Supplementary figures and images for: Identification of a new mechanism for targeting myosin II heavy chain phosphorylation by Dictyostelium myosin heavy chain kinase B
Source: BMC Res Notes. 2010 Mar 3;3:56. doi: 10.1186/1756-0500-3-56 (PMC2838905; doi:10.1186/1756-0500-3-56)

## Slide 1
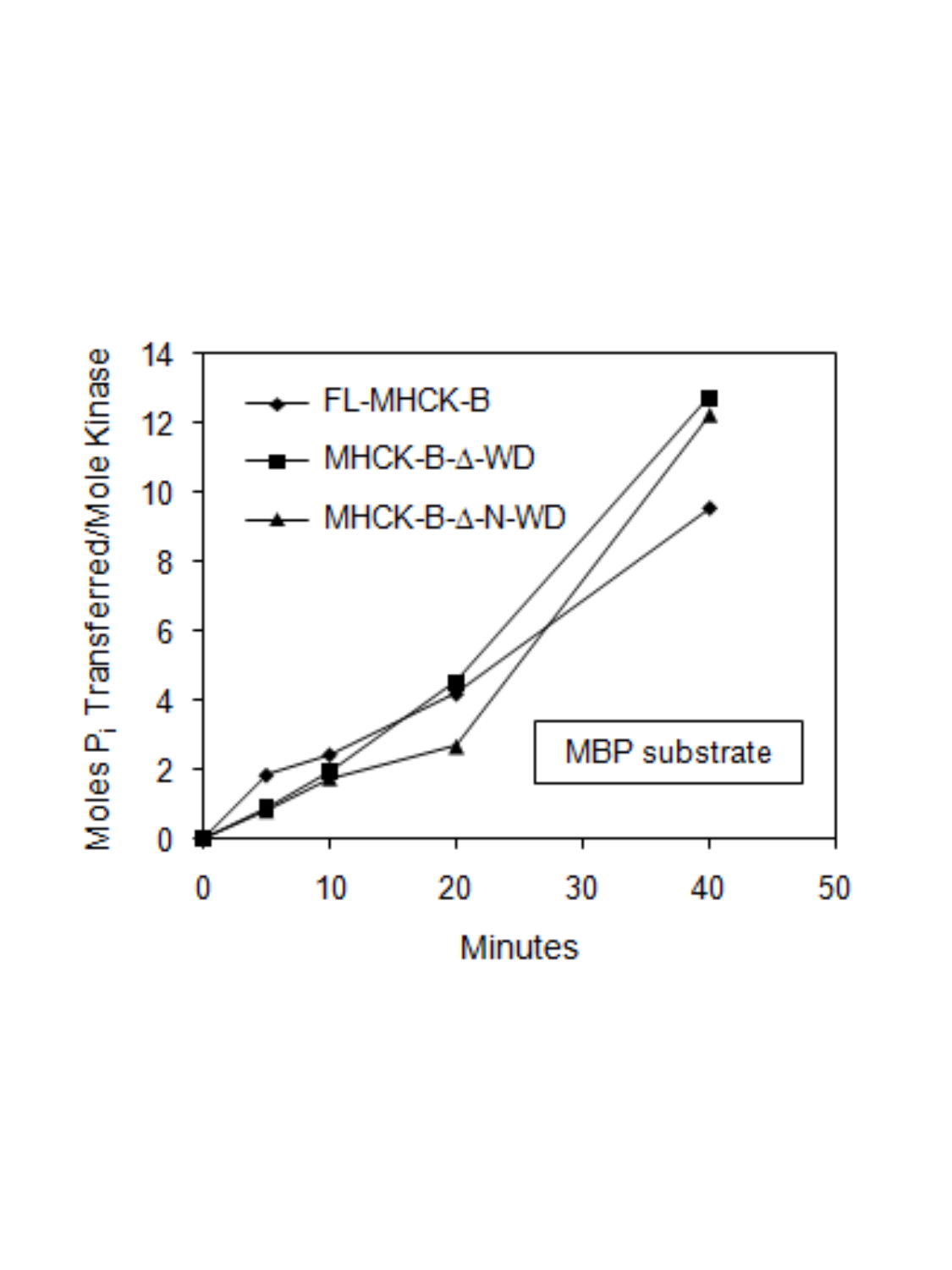

Supplement: Additional file 1 — Analysis of MHCK-B truncations for phosphorylation of myelin basic protein. Plots of myelin basic protein phosphorylation by full-length MHCK-B, MHCK-B-Δ-WD, and MHCK-B-Δ-N-WD over time. [file 1756-0500-3-56-S1.PPT]

## Slide 1
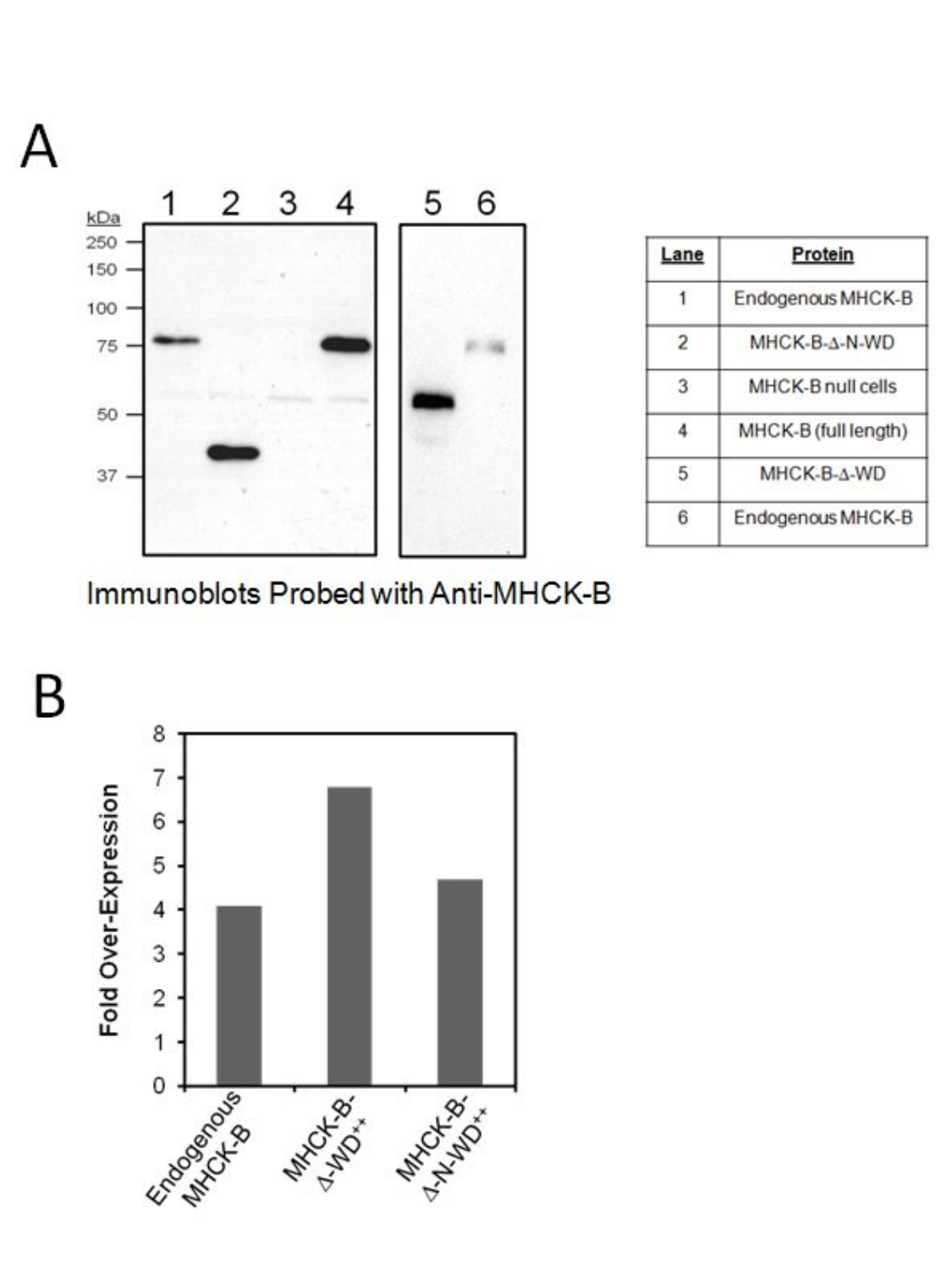

Supplement: Additional file 2 — Analysis of the expression levels of MHCK-B, MHCK-B-Δ-WD, and MHCK-B-Δ-N-WD in Dictyostelium cells. Immunoblots of cell lysates from AX2 cells (endogenous MHCK-B) and cells over-expressing MHCK-B, MHCK-B-Δ-WD, or MHCK-B-Δ-N-WD. Bar graph of the level of over-expression as determined by densitometric analysis of bands in the immunoblots. [file 1756-0500-3-56-S2.PPT]

## Slide 1
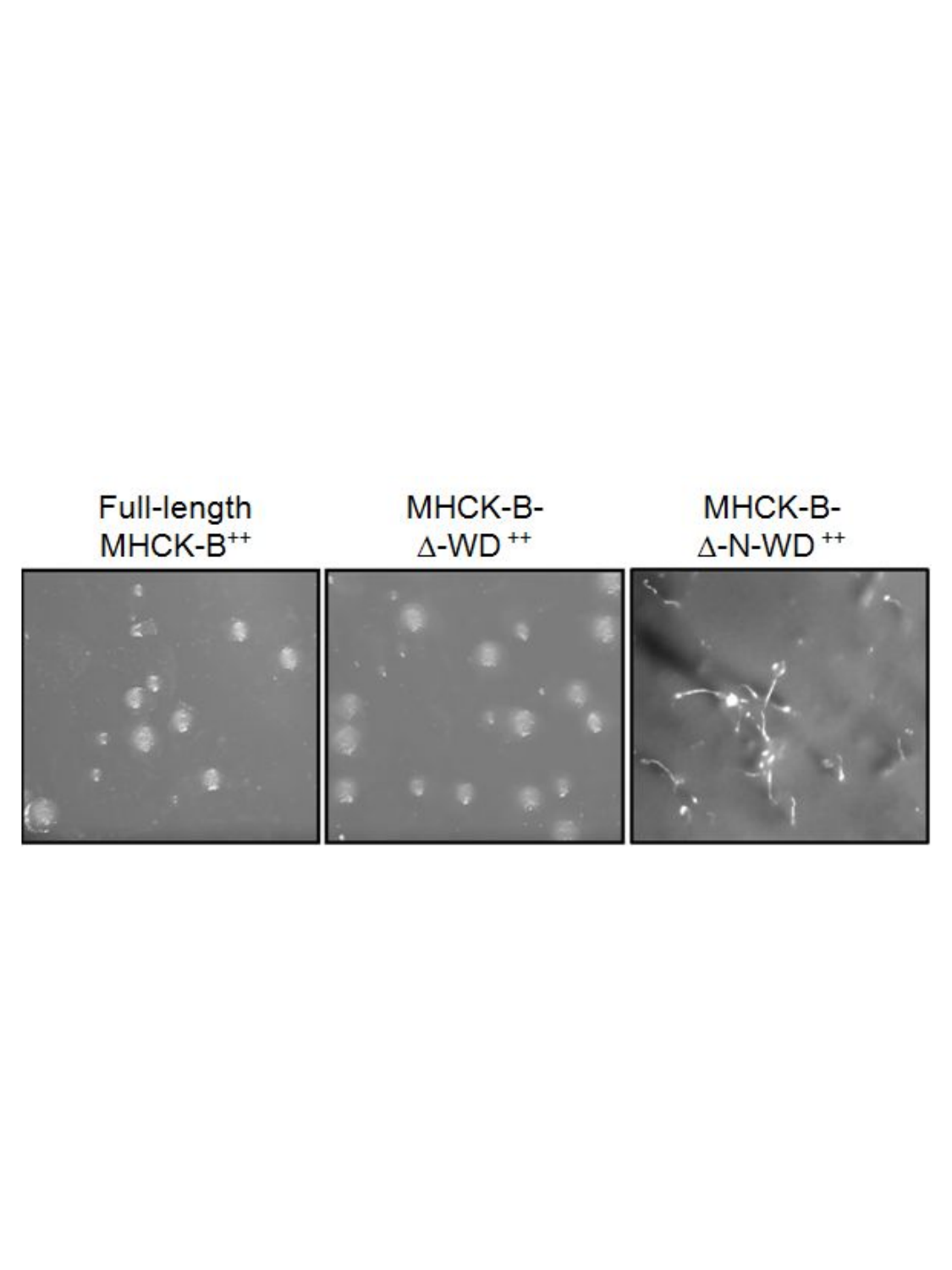

Supplement: Additional file 3 — Multicellular development of cells over-expressing full-length MHCK-B, MHCK-B-Δ-WD, or MHCK-B-Δ-N-WD. Digital images of the progress of Dictyostelium multicellular development after five days under starvation conditions. [file 1756-0500-3-56-S3.PPT]
